# Genetic Profiling and frequencies of Modifiers in Transfusion-Dependent Thalassemia

**DOI:** 10.12669/pjms.41.9.11571

**Published:** 2025-09

**Authors:** Gulrukh Sohail, Abid Sohail Taj, Arshi Naz, Muhammad Tariq Masood Khan

**Affiliations:** 1 Gulrukh Sohail, MBBS, M. Phil District Specialist (Pathology), Moulvi Ameer Shah Memorial Hospital, Peshawar, Pakistan; 2 Abid Sohail Taj, MBBS, Ph. D, MRCPath Consultant Clinical Hematologist, Peshawar General Hospital, Peshawar, Pakistan; 3 Arshi Naz, Ph. D, ISTH fellow Royal Free, UK HEC Approved Supervisor/Assistant Professor, Dept. of Pathology, Liaquat University of Medical & Health Sciences, Jamshoro, Pakistan; 4 Muhammad Tariq Masood Khan, MBBS, PhD Associate Professor (Hematology), Pak International Medical College, Peshawar, Pakistan

**Keywords:** Ameliorating factors, Genetic modifiers: polymorphism, Transfusion dependent thalassemia, α mutations, β mutations, *BCL11A*, *Xmn-1*

## Abstract

**Background & Objective:**

Thalassemia is a genetic blood disorder primarily influenced by β-globin gene mutations and various genetic modifiers. In Pakistan, especially among transfusion-dependent thalassemia (TDT) patients, data on these modifying factors remain limited, hindering accurate diagnosis and tailored management. This study aimed to reassess the diagnostic classification and determine the frequency of genetic determinants specifically alpha-thalassemia deletions and key polymorphisms (BCL11A and Xmn1-HBG2) associated with milder clinical phenotypes in multi-transfused thalassemia patients from the local population.

**Methodology:**

This descriptive cross-sectional study was conducted over a six-month period, from January to June 2019 and included 54 TDT patients aged up to 15 years from the Fatimid Foundation, Peshawar. Genetic analyses were performed for polymorphisms at Xmn-1-HBG2 and BCL11A, as well as for two alpha (α) and thirteen common beta (β) gene alterations. Utilizing the PureLinkTM Genomic DNA Kit, DNA was retrieved. The ARMS technique was used to identify β-thalassemia mutations, whereas Gap-PCR was used to identify α-thalassemia deletions. For Xmn-1 genotyping, we used RFLP-PCR, while ARMS-PCR was used to assess BCL11A polymorphisms.

**Results:**

All patients had either homozygous or compound heterozygous mutations in the β-globin gene. Of them, 38 patients had BCL11A polymorphisms, two had Xmn-1-HBG2 polymorphisms and 11 patients had a co-existing heterozygous α (3.7 kb) deletion. Homozygous Fr 8-9 was the variant with the most common mutation, occurring in 19 (35.2%) cases. Isolated β-globin gene mutations were found in just 13 cases. Together with the underlying β-globin mutation, 85.2% patients had an additional ameliorating genetic component (a BCL11A polymorphism, Xmn-1-HBG2 polymorphism, or α-globin gene mutation).

**Conclusion:**

Transfusion-dependent β-thalassemia patients in Peshawar Region often have co-existing genetic modifiers, resulting in a milder phenotype. Screening for these modifiers is recommended for specialized treatment in these children.

## INTRODUCTION

Thalassemia represents the most prevalent hereditary blood disorder worldwide, encompassing a group of inherited conditions caused by defects in hemoglobin structure or synthesis. As the most common monogenic disorder globally, thalassemia contributes significantly to morbidity and mortality across diverse populations.^1^,^2^

Thalassemia’s are characterized by a quantitative defect in hemoglobin synthesis, with β-thalassemia specifically arising from inherited mutations in the β-globin gene. This disease includes a wide range of hemoglobin abnormalities characterized by decreased production of β-globin chains.^2^ Approximately 80 to 90 million individuals, representing 1.5% of the global population, are carriers of β-thalassemia. In Pakistan, the carrier prevalence is around 5-8%, leading to an estimated 5,000-9,000 annual birth with β-thalassemia major (β-TM),^3^ which places a substantial burden on the healthcare system in this low- and middle-income nations. High rates of consanguineous marriages further concentrate defective genes within families, contributing to an increased disease incidence.^1^-^4^

Beta-thalassemia, characterized by reduced β-globin chain synthesis in hemoglobin^2^, involves over 200 identified mutations, resulting in substantial genotypic and phenotypic diversity.^5^ Clinically, β-thalassemia is categorized into minor (normal Hb level or mild anemia with no transfusion), intermedia (severe to mild anemia without regular transfusions) and major (severe anemia requiring lifelong transfusions), based on genetic mutations and symptom severity.^1^,^3^,^6^ Point mutations in the β-globin gene, which affect transcription, translation and splicing, are the primary cause of molecular lesions in β-thalassemia. In some cases, β-globin (HbB) gene deletions on chromosome 11 are also involved. While approximately 90% of aberrant β-globin alleles are caused by 20 mutations, phenotypic variations across people with the same genotype cannot be entirely explained by genotypic variability alone.^7^,^8^ Research has thus focused on identifying genetic modifiers that affect disease severity to support targeted therapies. β-thalassemia severity varies due to bi-allelic inheritance of β-globin gene copies on each chromosome 11 and diverse mutations classified as β+ (reduced production) or β° (absent production).^8^

In β-thalassemia, genetic modifiers at primary, secondary and tertiary levels significantly impact clinical severity by influencing globin chain synthesis and disease complications.^6^ Primary modifiers directly impair β-globin chain synthesis, leading to absent or reduced globin production. Secondary modifiers alleviate globin chain imbalance through mechanisms like α-thalassemia co-inheritance, increased γ-chain production and polymorphisms that enhance fetal hemoglobin (HbF) production, such as *Xmn-1* and *BCL11A* variants.^1^,^9^ Although they have no direct role in globin synthesis, tertiary modifiers affect genes linked to iron absorption, bilirubin metabolism, bone health, cardiovascular risk and infection receptivity, which in turn affects disease implications.^10^ The type of pathogenic mutation and the capacity for α- and γ-globin chain production are key determinants of disease expression.^11^

Better understanding of these genetic modifiers can substantially impact therapeutic management.^12^ Conventional hemoglobin studies primarily detect β-globin abnormalities, often overlooking α-globin disorders and genetic modifiers. Comprehensive genetic analysis remains uncommon, limiting the understanding of modifying factors. This study aimed to reassess the diagnostic classification and determine the frequency of genetic determinants specifically alpha-thalassemia deletions and key polymorphisms (BCL11A and Xmn1-HBG2) associated with milder clinical phenotypes in multi-transfused thalassemia patients from the local population.

## METHODOLOGY

This descriptive cross-sectional study was conducted over six months, from January to June 2019 and included 54 transfusion-dependent thalassemia (TDT) patients aged 15 years or older from the Fatimid Foundation, Peshawar. The guardians were informed about the objectives and benefits of the study and consent was acquired. Clinical and demographic information was gathered using a standard form. Samples of blood were examined and kept for later study at the National Institute of Blood Diseases in Karachi. Whole blood was used to extract genomic DNA via the Pure LinkTM Genomic DNA Kit.

### Ethical Approval:

The KMU Ethics Board accepted the study (Ref. No.: DIR/KMU/-EB/MC/000143; Dated: May 25, 2015).

The β-thalassemia mutations were analyzed using the ARMS PCR technique.^13^ Initially, the screening targeted the five most common mutations found in the local population, with subsequent rounds addressing less frequent and rare variants. Three sets of multiplex primers were utilized, each at a final concentration of 5 pM/μL which included both control primers and primers for common mutations. The PCR reaction mixture consisted of 10 μl of 1X PCR buffer, 1 μl of primer mix (1, 2, or 3), 0.2 μl of Taq polymerase and 2.5 μl of genomic DNA. This mixture was processed in an ABI® Veriti 96-well thermocycler under the following conditions: initial denaturation at 95°C for five minutes, followed by 25 cycles of denaturation at 94°C for one minute, annealing at 65°C for one minute and extension at 72°C for five minutes.

The amplified products were stored at 4°C. α-thalassemia deletions, specifically the -α3.7 kb deletion, were detected using Gap-PCR.^14^ The α-globin gene was amplified with forward primer C10 and reverse primers C2 and C3, targeting homologous Y regions and 3’ non-coding regions of the α1 and α2 genes. The 30 μL PCR mix contained 0.3 μg genomic DNA, 7.5 pM primers, 200 μM dNTPs, 1.9 mM MgCl2, 10% DMSO, 0.75 U Taq polymerase and 1x Taq buffer. The PCR was performed on an ABI Veriti® thermocycler with initial denaturation at 94°C for 10 minutes, followed by 30 cycles of 94°C for one minute, 52°C for one minute and 72°C for 90 seconds, with a final extension at 72°C for 10 minutes. PCR products were visualized on a 2% agarose gel stained with ethidium bromide, using a One kb DNA ladder.

The α4.2 deletion was identified using GAP-PCR, with the same cycling conditions as the α3.7 kb deletion. The reaction involved GAP primers (forward and reverse) at the α2 cap site and control primers. The presence of a 4.5 kb deletion generated a 1.319 kb band, while normal primers generated a 1 kb band. Xmn-1 genotyping (-158 Gγ C-T polymorphism) was done by RFLP-PCR 1 to amplify a 641-bp fragment around the -158 C-T polymorphism in the Gγ gene. Primers were purchased from Integrated DNA Technologies (USA). The thermal cycling included denaturation at 95°C for one minute, annealing at 60°C for one minute and extension at 72°C for five minutes. After amplification, a final extension was performed at 37°C with 10 units of PdmI (Xmn-1) restriction enzyme. PCR products were analyzed on a 2% agarose gel.

The BCL11A gene’s rs4671393 (A/G) SNP was identified using ARMS-PCR.^15^ A 548 bp fragment was amplified under the following conditions: 95°C for initial denaturation, followed by 40 seconds at 94°C for denaturation, 30 seconds at 62°C for annealing and 40 seconds at 72°C for extension, with a final extension at 72°C for five minutes. PCR products were separated by electrophoresis on a 2% agarose gel. Primers used included those for detecting the α 3.7 kb deletion (forward C10 and reverse C2 and C3) and for the α 4.2 kb deletion (forward and reverse primers targeting the α2 cap site with control primers). Xmn-1 genotyping used primers for the Gγ gene and BCL11A SNP genotyping was conducted with primer pairs specific to the rs4671393 (A) and (G) variants.

### Statistical analysis:

It was conducted using SPSS version 21, employing descriptive measures including frequencies, percentages, averages and standard deviations. The associations between categorical variables were assessed through Fisher’s exact test and X^2^ statistics, with the threshold for statistical significance established at α = 0.05.

## RESULTS

Among the 54 patients analyzed for mutations in the α- and β-globin loci and polymorphisms in BCL11A and Xmn-1, the cohort comprised 26 females (48.1%) and 28 males (51.9%). The average age of the participants was 11.0 ± 3.70 years, with ages ranging from three to 15 years. The age at diagnosis ranged from neonatal to two years of age. Upon diagnosis, the average total hemoglobin (Hb) level was found to be 6.202 ± 1.5 g/dL. Initial Hb measurements were taken from post-transfusion samples of eight patients. The diagnoses were established by integrating contextual factors, including a documented family history of thalassemia and the identification of thalassemia traits in both parents. Within this cohort, HbF levels exhibited a wide range, spanning from 5.1% to 60%, whereas in other individuals, levels were significantly higher, ranging between 70.0% and 98.5%.

Hepatomegaly and/or splenomegaly were identified in 23 patients (42.6%). Of these, three patients (5.6%) presented with isolated hepatomegaly, four (7.4%) exhibited isolated splenomegaly and the majority, 16 patients (69.5%), demonstrated concurrent enlargement of both the liver and spleen. All participants in the study received regular transfusions of packed red blood cells. Of these, 22 patients (40.7%) received transfusions every 10 days, while the majority (28 patients, 51.9%) had transfusions at intervals of 11 to 20 days. Four patients received transfusions at intervals of 21 to 30 days. Over the enrollment year, the total number of blood transfusions was documented, with participants receiving an average of 27 ± 11.1 transfusions each.

**Fig.1:**
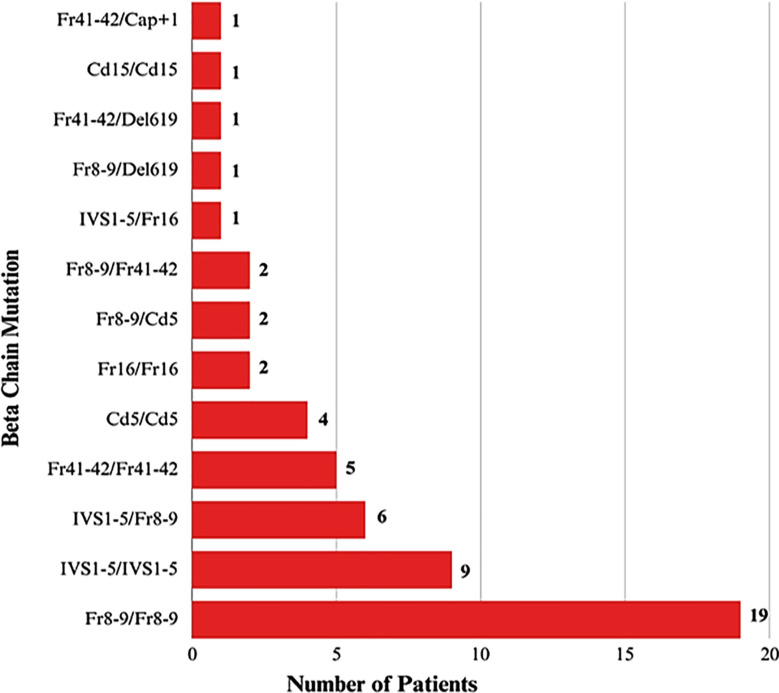
Frequencies of various β- globin gene mutations in study patients.

The study cohort had an average serum ferritin level of 7231.13 ± 5358.5 ng/ml; with levels ranging between 609.48 and 29793.28 ng/ml. Adequate chelation therapy was provided to only two patients; while the majority (52 patients, 96.3%) received insufficient iron chelation. A total of 54 patients underwent screening for mutations in the β-globin gene allele through allele-specific PCR, with particular attention to variants that have been previously reported in the local population. Of these, 37 patients displayed a homozygous β°/β° genotype and 17 were identified as compound heterozygotes (β+/β+ or β°/β+). The Fr8-9 mutation was notably the most common variant found (Fig.1).

The study cohort was further analyzed for locally prevalent α-thalassemia mutations. Heterozygous αα/-α3.7 deletion was identified in 9(16.66%) patients, while one patient carried the homozygous -α3.7/-α3.7 mutation and another presented with the αα/ααα anti3.7 mutation. All participants were also screened for BCL11A polymorphisms, revealing that 38(70.4%) patients carried the G>A polymorphism, whereas 16(29.6%) patients were negative for this variant. In contrast, Xmn-1 polymorphism was found to be rare, with only 2(3.7%) patients exhibiting heterozygosity, while 52(96.3%) patients lacked the polymorphism. Among the 37 patients with the β°/β° genotype, 25 carried the BCL11A polymorphism, eight had the co-existing -α3.7 deletion and only one displayed the Gγ-158 Xmn-1 polymorphism [+/-]. In contrast, none of the patients with the β+/β+ genotype exhibited the -α3.7 deletion or the Xmn-1 polymorphism, though seven were carriers of the BCL11A polymorphism. Of the eight patients with the β°/β+ genotype, six carried the BCL11A polymorphism, three were heterozygous for the -α3.7 deletion and one showed the Gγ-158 Xmn-1 polymorphism [+/-]. (Table-I).

**Table-I:** Frequencies of various genetic modifying factors among study patients.

Genotype	Alpha Globin Genotype	Xmn-1 Polymorphism	BCL11A Polymorphism	Total Detected	Total Not Detected	Total
βo/βo	αα/αα	[-/-]: 28, [+/-]: 1, [+/+]: -	Detected: 20	20	9	58
	αα/-α³·⁷	[-/-]: 6, [+/-]: -, [+/+]: -	Detected: 3	3	3	12
	-α³·⁷/-α³·⁷	[-/-]: 1, [+/-]: -, [+/+]: -	Detected: 1	1	-	2
	αα/αααanti³·⁷	[-/-]: 1, [+/-]: -, [+/+]: -	Detected: 1	1	-	2
Subtotal		Total [-/-]: 36, [+/-]: 1	Total Detected: 25	25	12	74
βo/β+	αα/αα	[-/-]: 5, [+/-]: -, [+/+]: -	Detected: 3	3	2	10
	αα/-α³·⁷	[-/-]: 2, [+/-]: 1, [+/+]: -	Detected: 3	3	-	6
Subtotal		Total [-/-]: 7, [+/-]: 1	Total Detected: 6	6	2	16
β+/β+	αα/αα	[-/-]: 9, [+/-]: -, [+/+]: -	Detected: 7	7	2	18
Subtotal		Total [-/-]: 9, [+/-]: -	Total Detected: 7	7	2	18
Grand Total		Total [-/-]: 52, [+/-]: 2	Total Detected: 38	38	16	106

About 85.2% patients exhibited ameliorating genetic factors. Comparative analysis of various clinical parameters with these genetic modifiers revealed that 56.5% of patients with ameliorating factors (n=26) were diagnosed after five months of age, while all patients without these factors were diagnosed earlier. In patients with ameliorating factors, 60.9% exhibited moderate anemia versus 39.1% with severe anemia; conversely, 87.5% of those without ameliorating factors presented with severe anemia. A significant association between hemoglobin levels and ameliorating factors was observed (p=0.019).

Transfusion frequency varied significantly; patients lacking ameliorating factors required transfusions every 10 days, while those with factors had extended intervals (p=0.001). Furthermore, 76.1% of patients with ameliorating factors exhibited HbF levels below 90%, revealing a significant association (p=0.009) with milder clinical presentations (Table-II).

**Table-II:** The relationship between genetic modifiers and clinical characteristics in the study cohort.

Parameters	Ameliorating factors			P-value
	Yes, n (%)	No, n (%)	Total	
***Diagnostic age***				
≤5 months	20(43.48)	8(100)	28(51.82)	0.015
5.1-11 months	22(47.83)	0	22(44.74)	
11.1-24 months	4(8.70)	0	4(7.41)
***Hb at diagnosis***				
Severe anemia (<6 g/dl)	18(39.13)	7(87.50)	25(46.30)	0.019
Moderate anemia (6-10 g/dl)	28(60.87)	1(12.50)	29(53.70)
***Transfusion Interval***				
≤10 days	16(34.78)	8(100)	24(44.44)	0.001
11-20 days	26(56.52)	0	26(48.15)	
21-30 days	4(8.70)	0	4(7.41)	
***Visceromegaly***				
Yes	23(50.00)	7(87.50)	30(55.56)	0.063
No	23(50.00)	1(12.50)	24(44.44)	
***HbF at presentation***				
≤ 90%	35(76.09)	2(25.00)	37(68.52)	0.018
>90%	11(23.91)	6(75.00)	17(31.48)	

Yes: α-globin gene mutation, Xmn-1-HBG2, or BCL11A polymorphism; No: primary β-globin mutation.

## DISCUSSION

Thalassemia, a prevalent monogenic disorder, presents with a broad phenotypic spectrum.^1^ In addition to impairing the hematopoietic system, it significantly impacts multiple organ systems, leading to various comorbidities and complexity.

This study analyzed 54 transfusion-dependent thalassemia (TDT) patients aged 3-15 years, with a nearly equal gender distribution. The predominant genetic determinant was homozygous or compound heterozygous inheritance of β° mutations (β°/β°), with Fr8-9 being the most frequent (35.2%), followed by IVS1-5 (16.7%), Fr41-42 (9.3%), Cd-5 (7.4%) and IVS1-5/Fr8-9 compound heterozygosity (11.1%). Genetic modifiers, including BCL11A polymorphisms and α-thalassemia deletions, were present in 85.2% of cases. These modifiers were significantly associated with later age at diagnosis, milder anemia, reduced transfusion requirements and lower HbF levels. The α3.7 deletion was the most common α-globin mutation, observed in 11 patients. Despite high serum ferritin levels and suboptimal chelation in most participants, the presence of ameliorating genetic factors was linked to less severe clinical phenotypes. These findings are consistent with regional data from Iran,^16^ where β° mutations are also more prevalent than β° mutations in thalassemia intermedia. Co-inheritance of α-thalassemia remains a well-recognized factor in mitigating disease severity in β-thalassemia patients.^17^

In this study, the Xmn-1 polymorphism (+/-) was found in just two patients (3.7%), one exhibiting the β°/β° genotype and the other the β°/β+ genotype. These findings align with those of Verma et al., who reported a similar distribution of Xmn-1 polymorphism among 325 thalassemia intermedia (TI) patients across diverse ethnic groups in the Mediterranean and Asia. Their study concluded that Xmn-1 is the most prevalent genetic modifier in cases involving β° mutations, but not in those with β+ mutations.^18^

In the current study, a significant majority of patients (n = 38, 70.3%) exhibited BCL11A polymorphisms. This result is consistent with earlier studies on the Sardinian population, which found that variations at the BCL11A locus have a significant effect on HbF levels, leading to a positive modification of the clinical phenotype of β-thalassemia.^19^ These differing distributions may reflect population-specific genetic backgrounds and modifier patterns, suggesting that BCL11A plays a more prominent modulatory role than Xmn-1 in this Pashtun cohort. This highlights the importance of local genetic studies in understanding variability in clinical severity among thalassemia patients, even within broadly similar mutation spectra.

The findings of this study show that 31.4% of patients exhibit β+ alleles in either homozygous or compound heterozygous forms, which are associated with milder phenotypic expressions. Additionally, co-inheritance of the α3.7 kb deletion in 20.3% of the cohort emerges as a significant ameliorating factor. Furthermore, 3.7% of patients exhibit heterozygous Xmn-1 polymorphism, contributing to a less severe disease phenotype. Notably, 70.4% of participants harbor co-inherited BCL11A polymorphism, a recognized independent ameliorating factor. Although the presence of genetic modifiers suggests a trend toward milder disease, the continued need for transfusions indicates a more severe clinical presentation. This discrepancy highlights the influence of both genetic and non-genetic factors such as mutation type, healthcare access and timing of diagnosis on disease severity.

### Limitations:

The relatively small sample size limits the generalizability of the findings and reduces statistical power. Additionally, diagnostic misclassification may also have occurred, as intercurrent infections and poor nutritional status in thalassemia intermedia patients can mimic the clinical features of thalassemia major, especially in resource-limited settings like Pakistan. These factors highlight the need for comprehensive clinical and genetic evaluation to improve diagnostic accuracy and patient management in low- and middle-income countries.

## CONCLUSIONS

Genetic ameliorating variables were found to be much more common in study patients with transfusion-dependent β-thalassemia. A milder conditions phenotype is linked to these characteristics, which calls for specialized management strategies. It is recommended that genetic screening for these factors, along with an evaluation of their responses to various treatment strategies in larger cohorts, be undertaken to establish definitive clinical guidelines.

### Author’s contributions:

**GS and AST:** Conception, ideas, design & data collection.

**GS and AN:** Data analysis and interpretation/results.

**GS, TMK and AST:** Manuscript drafting and writing.

**GS, TMK, AST and AN:** Language editing, critical revision. All authors have read and approved the study.

The principal investigator is responsible and accountable for the accuracy or integrity of the work.
